# Retraction: Commentary: computational analysis for ERAS and other surgical processes: commentary from clinical perspective

**DOI:** 10.3389/fsurg.2023.1237617

**Published:** 2023-06-19

**Authors:** Frontiers Editorial Office

A Retraction of the Original Research Article Commentary: Computational Analysis for ERAS and Other Surgical Processes: Commentary From Clinical Perspective By Mills H, Acquah R, Tang N, Cheung L, Klenk S, Glassen R, Pirson M, Albert A, Hoang DT and Van TN (2022). Front. Surg. 9:946963. doi: 10.3389/fsurg.2022.946963.

Following publication, the publisher uncovered evidence that several false identities were listed as authors for this article. During this investigation, the publisher was unable to confirm the institutions of the authors Hilla Mills, Ronald Glassen, Susanne Klenk, Nova Tang, or Luke Cheung. This investigation was conducted in accordance with Frontiers' policies and the Committee on Publication Ethics (COPE) guidelines.

The investigation also uncovered concerns about the presentation and validity of the data in the article.

This retraction was approved by the Chief Editors of Frontiers in Surgery and the Chief Executive Editor of Frontiers.

The authors did not respond to contact regarding this retraction.

